# Psychosocial Working Conditions Play an Important Role in the Return-to-Work Process After Total Knee and Hip Arthroplasty

**DOI:** 10.1007/s10926-021-10006-7

**Published:** 2021-09-28

**Authors:** Tamara Kamp, Sandra Brouwer, Tjerk H. Hylkema, Jan van Beveren, Paul C. Rijk, Reinoud W. Brouwer, Martin Stevens

**Affiliations:** 1 Department of Orthopedics, University Medical Center Groningen, University of Groningen, P.O. Box 30.001, 9700 RB Groningen, The Netherlands; 2 Department of Health Sciences, Community and Occupational Medicine, University Medical Center Groningen, University of Groningen, Groningen, The Netherlands; 3 Department of Orthopedics, Röpcke-Zweers Hospital Hardenberg, Hardenberg, The Netherlands; 4 Department of Orthopedics, Medical Center Leeuwarden, Leeuwarden, The Netherlands; 5 Department of Orthopedics, Martini Hospital Groningen, Groningen, The Netherlands

**Keywords:** Knee prothesis, Hip prothesis, Return to work, Workplace, Work characteristics, Psychosocial working conditions, Physical work factors

## Abstract

*Purpose* Both personal and work-related factors affect return to work (RTW) after total knee arthroplasty (TKA) and total hip arthroplasty (THA). Little is known about work-related factors associated with the recovery process. This study aimed to determine which work-related factors are associated with time to RTW for both TKA and THA patients. *Methods* A prospective multicenter survey study was conducted that included patients aged 18–63, had a paid job and were scheduled to undergo primary TKA/THA. Surveys were completed preoperatively, 6 weeks, and 3, 6, and 12 months postoperatively, and included four domains of work-related factors: work characteristics, physical working conditions, psychosocial working conditions and work adjustments. Control variables included age, sex, education, and comorbidity. Time to RTW was defined as days from surgery until RTW. Multivariate linear regression analyses were conducted separately for TKA/THA patients. *Results* Enrolled were 246 patients (n = 146 TKA, n = 100 THA, median age 56 years, 57% female). Median time to RTW was 79 days (IQR 52.0–146.0). Mainly physical tasks (TKA: B 58.2, 95%CI 9.5–106.8; THA: B 52.1, 95%CI 14.1–90.2) and a combination of physical and mental tasks (TKA: B 50.2, 95%CI 6.4–94.0; THA B 54.0, 95%CI 24.2–83.7) were associated with longer time to RTW after both TKA and THA. More possibilities for personal job development (B − 12.8, 95%CI − 25.3–0.4) and more work recognition (B − 13.2, 95%CI − 25.5 to − 0.9) were significantly associated with shorter time to RTW after TKA. Higher quality of supervisor leadership (B − 14.1, 95%CI − 22.2 to − 6.0) was significantly associated with shorter time to RTW after THA. *Conclusion* The findings of this study stress the importance of psychosocial working conditions, besides type of job tasks, in RTW after TKA/THA. Further research on work-related factors is needed, as arthroplasty is being performed on an increasingly younger population of knee and hip OA patients for whom participating in work is of critical importance.

## Introduction

Osteoarthritis (OA) is one of the most common reasons for chronic musculoskeletal pain [1]. It is a highly prevalent chronic joint disease that affects about one in eight adults worldwide [2]. Among working-age individuals OA is one of the leading causes of disability [3], and is strongly associated with reduced productivity among working individuals [4]. Due to aging and the rise in obesity in Western countries, OA’s burden and the associated disability among the working-age population will become substantial in the coming years [5, 6].

Total knee arthroplasty (TKA) and total hip arthroplasty (THA) are effective procedures to reduce pain and improve function in patients with hip or knee OA [7, 8]. In the Netherlands 25,566 primary TKAs and 31,594 primary THAs were performed in 2019 [9], and an expected 57,900 and 51,680 patients will undergo TKA or THA, respectively, by 2030. The greatest spike in TKA and THA is seen in patients of working age. The number of TKA and THA patients below age 65 already tripled between 1995 and 2003 in the Netherlands, and this number is expected to rise further [10].

Due to the increasing numbers of TKAs and THAs among working-age patients and the rising retirement age, more patients have to return to work (RTW) after surgery [10–17]. While the majority of patients do return to work (71–83% after TKA, 68–95% after THA), time to RTW varies [18]. Both personal and work-related factors associated with (time to) RTW after TKA or THA have been found [18–20]. However, most studies focus on personal factors [21–23] and only a limited number have aimed to investigate how work-related factors may affect the RTW process in TKA or THA patients [20, 24, 25].

Work-related factors influencing RTW can be roughly subdivided into four domains: workplace characteristics (e.g. working hours, type of contract, tasks, job type), physical working conditions (e.g. standing, walking, sitting), psychosocial working conditions (e.g. work pace, role clarity, job satisfaction) and work adjustments (e.g. lighter duties, shorter hours, different workstation). Adverse physical and psychosocial working conditions are generally associated with premature exit from the labor force [26]. Work adjustments have been mentioned as a successful strategy to accommodate workers in returning to work and successfully perform their job tasks [27, 28].

In the orthopedic literature some studies found that physically demanding jobs may hinder RTW after TKA or THA [20, 21, 24]. It was also found that being self-employed facilitated RTW [20], that workers who perform knee-burdening work and identify their knee symptoms as work-related have high chances of not returning to work [24], and that workplace support and adaptation of the job role had a positive impact when patients did RTW [29, 30]. Moreover, job flexibility has been associated with earlier RTW [31]. Successful RTW after TKA or THA may benefit from workplace adaptations and accommodations [29] as well as handicap accessibility [20].

These results stress the importance of considering work-related factors in the timely referral to work-directed care of patients at risk for not returning to work after TKA or THA. However, given that those studies only include a limited number of work-related factors and mainly focus on a specific domain, relatively little is known about the influence of work-related factors on time to RTW taking into account all four work domains. Hence the aim of this study was to determine which work-related factors are associated with time to RTW after TKA or THA.

## Material and Methods

### Design and Procedure

A prospective multicenter survey study was conducted among patients who underwent TKA or THA for primary OA. This study was part of the “Work participation In Patients with Osteoarthritis” cohort (WIPO, Trial-ID NTR3497) [32–34]. Patients were recruited between March 2012 and July 2014 at the orthopedic departments of four Dutch medical centers: University Medical Center Groningen (tertiary university hospital), Martini Hospital Groningen (large teaching hospital), Medical Center Leeuwarden (large teaching hospital) and Röpcke-Zweers Hospital Hardenberg (general hospital), all in the northern Netherlands. Patients who were on a waiting list for TKA or THA were contacted by phone and invited to participate in the study. Preoperative questionnaires were filled in approximately one month before surgery. Postoperative follow-up data were collected at 6 weeks and 3, 6, and 12 months. If applicable, missing answers were added later to the questionnaire after contacting the patients by phone. Informed consent was obtained at baseline.

### Participants

Patients with knee or hip OA undergoing TKA or THA, preoperatively employed and aged 18–63 were included. Excluded were patients with insufficient knowledge of the Dutch language, those having undergone a unicompartimental knee arthroplasty or revision TKA or THA. A dropout was defined as a patient leaving the study preterm by not filling in one of the postoperative questionnaires for any reason.

### Measures

#### Time to Return to Work

Time to return to work was the outcome measure, defined as length of time (days) from surgery to RTW. RTW was defined as the first time participants partially or fully returned to work after surgery. RTW (yes/no) and the specific date was asked in the follow-up questionnaires at 6 weeks and 3, 6 and 12 months postoperatively.

#### Work-Related Factors

*Work characteristics* included questions about self-employment (yes/no), company size (number of employees: 1–9, 10–99, more than 100), contractual hours (h), working hours (h), type of job (executive/administrative/advisory/management/policy), and type of tasks (physical/mental/combination).

*Physical working conditions* were measured with a self-structured questionnaire by asking whether patients had to perform physical activities like standing, sitting, walking, kneeling or squatting (yes/no), and whether they perceived difficulties in performing physical work demands (9 items) due to impairing knee or hip problems (yes/no).

*Psychosocial working conditions* were measured using three domains from an adapted version of the short version of the Copenhagen Psychosocial Questionnaire II (COPSOQ-II). The domains included (1) demands at work, (2) work organization and job contents, and (3) interpersonal relations and leadership. The first domain included the dimensions quantitative demands, work pace and emotional demands, the second domain included the dimensions influence at work, possibilities for development, meaning of work and commitment to the workplace, and the third domain included the dimensions predictability, recognition (reward), role clarity, quality of leadership, social support from supervisor and social support from colleagues. Each dimension consisted of two questions.

All questions were scored on a five-point scale, ranging from never [1] to always [5], thus a higher score indicated a higher exposure. The total dimension score was calculated as the sum of scores of the questions within each dimension, thus scores could range from 2 to 10. The short version of the COPSOQ-II has been proven to be valid and reliable [35].

*Work adjustments* were assessed by asking “Were adjustments made to your work since your complaints?” (yes/no) and the follow-up question “Which adjustments have been made to your work since your complaints?”, with the following answer options: shorter working hours, fewer contractual hours, change of function, change of tasks, changed working hours, cessation of managerial duties, less demanding work, more frequent breaks, flexible schedule, workplace adjustments, additional employee who fulfills the tasks I cannot do, ergonomic adjustments. Multiple answers were allowed. We calculated a percentage of the total accommodations made and also created a dichotomous variable (yes/no). Patients receiving one or more work accommodations were coded as yes.

### Covariates

Data on the following sociodemographic data were collected: age, sex, living alone or with a partner, educational level (categorized into elementary, secondary and higher), being a wage earner (yes/no). Disease-related information was gathered by asking about type of arthroplasty (TKA or THA), body mass index (BMI) divided into normal (< 25) and overweight/obese (> 25), and comorbidity measured with a 27-item chronic conditions questionnaire [36]. Number of comorbidities was categorized into having no, one and two, or more than two comorbidities.

### Statistics

Descriptive statistics—mean (SD), n (%)—were used to describe baseline characteristics of the study population. Kaplan–Meier survival analysis was performed to calculate median time to RTW for the entire group and the subgroups (TKA and THA). Linear regression analysis was used to study the prognostic factors for time to RTW. First the association between time to RTW and each potential prognostic factor was univariately assessed. Age, sex, educational level and number of comorbidities were included as control variables [18, 25]. All prognostic factors with a p-value ≤ 0.20 in the univariate analyses were included in the model and analyzed using a backward stepwise selection method. Next, multiple regression analyses were performed in two models. For model 1 the analyses were performed in blocks: in block 1 workplace characteristics were added, in block 2 physical working conditions, in block 3 psychosocial working conditions and in block 4 work adjustments. For every block the factors were removed through backward stepwise selection until only variables below the cut-off value (p ≤ 0.20) remained. For model 2, the final model, all factors of the separate multivariate regressions that were below the cut-off value (p ≤ 0.20) were included, then removed through a backward stepwise selection method until a statistically significant final model remained. A p-value < 0.05 was considered statistically significant. Statistical analyses were performed with IBM Statistical Package for the Social Sciences (SPSS) version 25.0.

## Results

**Fig. 1:**
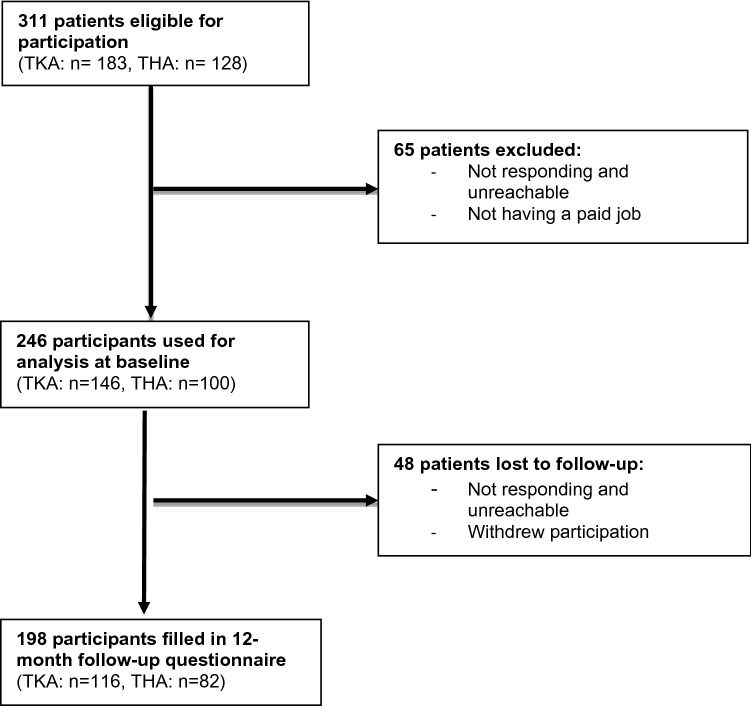
Flowchart study enrolment and follow-up

**Fig. 2:**
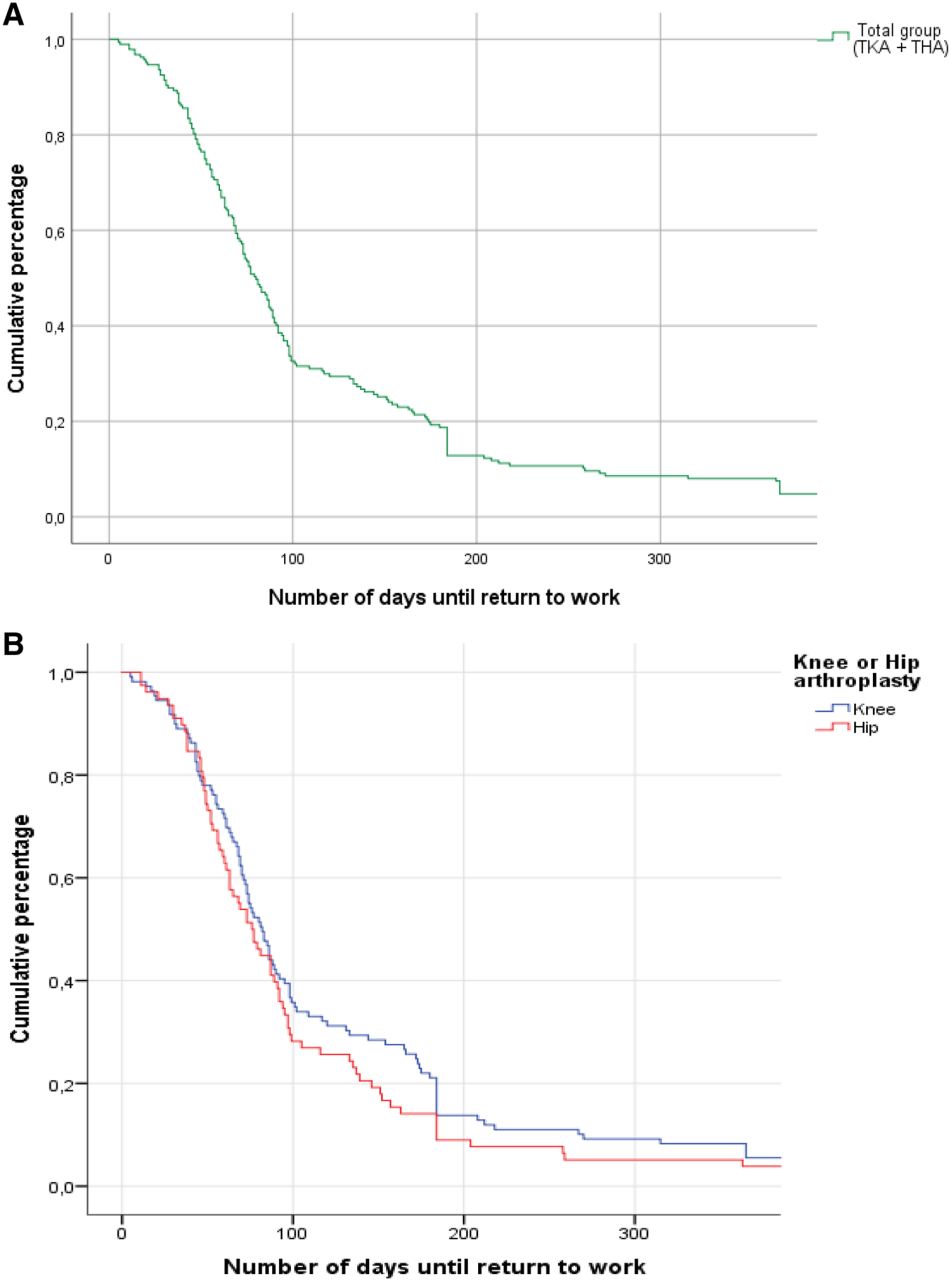
**a** Kaplan–Meier curve—cumulative percentage of RTW of the total group (TKA and THA). **b** Kaplan–Meier curve—cumulative percentage of RTW of the subgroups TKA and THA

Of the 311 patients who had undergone a primary TKA or THA, 246 (n = 146 TKA, n = 100 THA; response rate 79.1%) were included. Figure 1 is a flow chart showing the total number of patients at baseline and the drop-outs to follow-up. The characteristics of the study sample are presented in Table 1. Median age of the total patient group was 56 years (interquartile range (IQR) 51–59 years). The sample consisted of 43% (n = 107) men and 57% (n = 139) women, 59% (n = 146) TKA patients and 41% (n = 100) THA patients; 76% of workers had completed lower or secondary education and 21% higher education. Median time to RTW was 79 days (IQR 52.0–146.0) (Fig. 2a, b). Follow-up data at 12 months was available for 198 (80.5%) of the 246 patients enrolled. Patients who dropped out during the study did not differ from the study sample on any covariates. In total, 90.4% (9.6% partial, 80.8% full-time) of the patients returned to work within 12 months of surgery.

**Table 1:** Baseline study sample characteristics

Characteristics	Total (N = 246)	TKA (N = 146)	THA (N = 100)
Age (median, IQR)	56 (51–59)	56 (52 – 59)	56 (50 – 60)
Male/female [n (%)]	107 (43)/139 (57)	61 (42)/85 (58)	46 (46)/54 (54)
BMI (kg/m^2^) [n (%)]			
< 25	54 (22)	23 (16)	31 (31)
> 25	187 (76)	120 (82)	67 (67)
TKA/THA [n (%)]	146 (59)/100 (41)	–	–
Highest educational level [n (%)]			
Lower (elementary school, vocational education)	81 (33)	48 (33)	33 (33)
Secondary (high school, intermediate vocational education)	105 (43)	67 (46)	38 (38)
Higher (higher professional education, university)	52 (21)	27 (19)	25 (25)
Partner [n (%)]	224 (91)	132 (90)	92 (92)
Number of comorbidities [n (%)]			
None	20 (8)	8 (6)	12 (12)
One or two	138 (56)	77 (53)	61 (61)
More than two	87 (35)	61 (42)	26 (26)
Wage earner [n (%)]	133 (54)	75 (51)	58 (59)
Self-employed [n (%)]	31 (13)	19 (13)	12 (11)
Time to return to work (median, IQR)	79.0 (52.0–146.0)^a^	82.0 (55.0–172.5)^b^	76.5 (49.0–113.5)^c^

All numbers are represented as mean with standard deviation (SD) or numbers (n) and percentages (%)

^a^N = 198; ^b^N = 116; ^c^N = 82

### Univariate and multivariate analyses among TKA patients

In the univariate analyses five work characteristics (self-employment, contractual hours, working hours, type of job, type of tasks) and six physical working conditions (standing, sitting, walking, difficulty with sitting, difficulty moving more than 20 kg, difficulty with driving) were below the cut-off value and therefore used in the first multivariate analysis (Appendix). Of the psychosocial working conditions four variables were below the cut-off value (influence at work, possibilities for development, predictability, recognition). There was no association between receiving a work adjustment and time to return to work.

In the multivariate analysis two work characteristics (job type, type of tasks), two physical working conditions (work that demands walking, difficulty with sitting) and three psychosocial working conditions (influence at work, possibilities for development, recognition) were below p ≤ 0.20 (model 1). In the final model mainly physical tasks (B 58.2, 95%CI 9.5–106.8) and a combination of physical and mental tasks (B 50.2, 95%CI 6.4–94.0) were associated with longer time to RTW. More possibilities for development (B -12.8, 95%CI -25.3–0.4) and more recognition (B − 13.2, 95%CI − 25.5 to − 0.9) were significantly associated with shorter time to RTW (model 2).

### Univariate and multivariate analyses among THA patients

In the univariate analyses the work characteristics company size and type of tasks were below the cut-off value and therefore used in the first multivariate analysis (Appendix). Of the physical working conditions four variables (sitting, kneeling or squatting, difficulty working in an uncomfortable position, difficulty working in the same position for an extended period) were below the cut-off value (Appendix). Of the psychosocial working conditions four variables were below the cut-off value (quantitative demands, work pace, possibilities for development, quality of leadership; Appendix). There was no association between receiving a work adjustment or percentage of work adjustments and time to RTW.

In the multivariate analysis type of job tasks, two physical working conditions (kneeling or squatting and difficulty working in an uncomfortable position) and three psychosocial working conditions (possibilities for development, tempo work pace, quality of leadership) were below p ≤ 0.20 (model 1). In the final model mainly physical tasks (B 52.1, 95%CI 14.1–90.2) and a combination of physical and mental tasks (B 54.0, 95%CI 24.2–83.7) were significantly associated with longer time to RTW; higher quality of leadership (B − 14.1, 95%CI − 22.2 to − 6.0) was significantly associated with shorter time to RTW (model 2; Table 2).

**Table 2:** Multivariate regression analyses for the outcome RTW (days) after total knee arthroplasty (TKA) and total knee arthroplasty (THA)

Variables	TKA						THA					
	Model 1			Model 2			Model 1			Model 2		
	B	95% CI	P value	B	95% CI	P value	B	95% CI	P value	B	95% CI	P-value
Workplace characteristics												
Job type												
Executive (ref = Policy)	38.6	− 18.2–95.5	0.18	NS	NS	NS	NS	NS	NS	NS	NS	NS
Administrative (ref = Policy)	0.44	− 73.1–74.0	0.99	NS	NS	NS	NS	NS	NS	NS	NS	NS
Advisory (ref = Policy)	− 11.1	− 118.6–96.6	0.84	NS	NS	NS	NS	NS	NS	NS	NS	NS
Management (ref = Policy)	52.1	− 14.5–118.6	0.12	NS	NS	NS	NS	NS	NS	NS	NS	NS
Tasks												
Physical (ref = mental)	38.4	− 14.0–90.76	0.15	58.2	9.5–106.8	0.02*	54.4	13.5–95.3	0.01	52.1	14.1–90.2	0.01*
Both (ref = mental)	36.2	− 11.62–84.06	0.14	50.2	6.4–94.0	0.03*	49.7	17.2–82.3	0.00	54.0	24.2–83.7	0.00*
Physical working conditions												
Work demands walking (ref = no)	47.2	11.9–82–6	0.01	NS	NS	NS	NS	NS	NS	NS	NS	NS
Work demands kneeling/squatting (ref = no)	NS	NS	NS	NS	NS	NS	42.1	3.0–81.1	0.04	NS	NS	NS
Difficulty sitting (ref = no)	30.0	− 3.5–63.4	0.08	NS	NS	NS	NS	NS	NS	NS	NS	NS
Difficulty working in uncomfortable position (ref = no)	NS	NS	NS	NS	NS	NS	39.0	5.0–73.0	0.03	NS	NS	NS
Psychosocial working conditions												
Influence at work	− 7.3	− 17.9–3.2	0.17	NS	NS	NS	NS	NS	NS	NS	NS	NS
Possibilities for development	− 10.7	− 24.1–2.7	0.12	− 12.8	− 25.3–0.4	0.04*	− 7.3	− 17.5–2.9	0.16	NS	NS	NS
Recognition	− 15.6	− 27.4–3.8	0.01	− 13.2	− 25.5–0.9	0.04*	NS	NS	NS	NS	NS	NS
Tempo work pace	NS	NS	NS	NS	NS	NS	9.9	1.3–18.5	0.02	NS	NS	NS
Quality leadership	NS	NS	NS	NS	NS	NS	− 12.7	− 21.4–4.1	0.01	− 14.1	− 22.2–6.0	0.00*

Model 1 = blocks (workplace characteristics and physical working conditions, psychosocial working conditions, work adjustments)

Model 2 = total

Adjusted for age, sex, education, and number of comorbidities. *P < 0.05. *NS* not significant

## Discussion

This study aimed to investigate which work-related factors influence time to RTW after TKA or THA. We found that besides type of job tasks, the key factors were psychosocial working conditions for both groups, with some additional differences between TKA and THA patients in the type of psychosocial working conditions associated with time to RTW within 12 months of surgery.

Our findings about the role of psychosocial working conditions in RTW could not be compared to other studies on TKA or THA due to a lack of research investigating this influence. We found that possibilities for personal job development, more work recognition and high quality of supervisor leadership resulted in a significant shorter time to RTW. Comparison with other studies among workers with chronic diseases exposed similar findings [37–39].

Quality of leadership from the supervisor showed to enhance the likelihood to accommodate workers with back injuries and prevent prolonged work disability [38]. Possibilities for development, i.e. job control (including work autonomy), was evidenced as a strong facilitator of RTW among different population groups [37]. In line with our results, the importance of recognition, appreciation, good communication and genuine concern from the supervisor for RTW outcomes has been shown in different population groups [40, 41].

Although some studies among TKA or THA patients suggest that social support may result in better postoperative outcomes [30], we did not find a significant association between work-related social support from the supervisor and/or colleagues and time to RTW. Previous research indicates that supervisors’ support and leadership quality were effective in reducing sickness absenteeism and may play an important role in the RTW process [42].

Our findings showed that THA and TKA patients who perform mainly physical tasks and a combination of physical and mental tasks have a longer time to RTW compared to those in jobs with mental tasks. Other studies also found jobs with mainly physical tasks as an impeding factor for RTW [20, 21, 24, 43]. However, in contrast to a previous study among TKA patients [21] we did not find an association between physical working conditions and time to RTW in the multivariate model. This may suggest that psychosocial working conditions are more important for RTW after TKA or THA than physical working conditions.

We did not find an association between preoperative work adjustments and time to RTW either. In our study population only 27.3% of workers received work adjustments. Even though the number of work accommodations received is slightly higher than in previous research among TKA or THA patients (20%) [18], this may have influenced our outcome as we only investigated preoperative work accommodations. More accommodations can be expected to be made postoperatively. It has nonetheless been reported that workers who modified their responsibilities preoperatively are more likely to do so postoperatively [20].

In our study the majority of patients (89.7% TKA, 91.5% THA) returned to work postoperatively, which is in line with previous studies [20, 21, 44]. Furthermore, approximately half of our sample returned to work partially or fully within the first three months postoperatively, with a median RTW of 79 days (11.3 weeks), which is similar to the median time to RTW reported in literature [7, 20, 23, 45, 46].

### Strengths and limitations

A strength of this study is the prospective design with its relatively large number of patients, multiple follow-up moments and outcome measures up to 12 months postoperatively. This gave us the opportunity to examine specific time to RTW. Another strength is the representative sample of patients and therefore the generalizability of the results.

This study has also some limitations. Measurements were self-reported, therefore generally susceptible to the effects of reporting bias. Another limitation was a 20% (48/246 patients) dropout rate at 12 months follow-up. Comparison of non-responders at 12 months follow-up with responders revealed no significant differences in baseline characteristics. We only focused on the first time workers partially or fully returned to work, therefore not taking into account whether work absences recurred after the first RTW.

### Implications

Changing workforce dynamics and trends toward surgery at younger ages mean that these are important outcomes for clinicians to assess besides those of pain and function that are usually reported following TKA or THA. Information about the role of the work environment is also important for occupational and health practitioners as well as for employers toward understanding workers’ continued participation in employment after TKA or THA. So far most studies have focused on the impact of physical work-related factors on RTW [18]: our study shows that psychosocial working conditions may also play an important role in the RTW process after TKA or THA. The results of this study provide a number of factors (i.e. possibilities for personal job development, work recognition, quality of supervisor leadership) to facilitate RTW after TKA or THA that can be built into the design and implementation of effective RTW intervention programs. Previous studies among other populations (i.e. workers with mental disorders, back injuries, cardiovascular diseases and cancer) reported that these interventions should in particular target the employer to enhance RTW support for sick listed workers [37, 38, 41]. For example, supervisor training programs aiming to improve leadership [38] and interventions addressing organizational culture to facilitate employee’s needs (e.g. increase job control and recognition) [37, 41]. To our knowledge, this is the first quantitative study to extensively examine the role of psychosocial working conditions among this specific population. Further research is needed to confirm our findings. Additional research is needed to enrich future understandings of the contribution of work-related social support in TKA and THA patients and to explore whether work absences recur after the first RTW.

## Conclusion

The present study showed that, besides type of job tasks, psychosocial working conditions may play a key role in facilitating time to RTW after primary TKA or THA. Although some differences in factors were found between TKA and THA patients, our findings suggest overall that possibilities for personal job development, work recognition and quality of supervisor leadership are important factors toward RTW after arthroplasty. Further research on the role of physical and psychosocial working conditions as well as work adjustments is needed, as arthroplasty is being performed on an increasingly younger population of knee and hip OA patients for whom participating in work is of critical importance.

## Appendix

See Table 3.

**Table 3:** Univariate regression analyses for the outcome RTW (days) after total knee arthroplasty (TKA) and total hip arthroplasty (THA)

Variables	TKA			THA		
	B	95% CI	P value	B	95% CI	P value
Workplace characteristics						
Salaried (ref = Self-employed)	45.7	6.0 to 85.4	0.024*	25.9	− 18.5 to 70.3	0.25
Company size						
0–9 employees (ref ≥ 100)	− 4.4	− 44.2 to 35.4	0.83	− 35.0	− 74.5 to 4.5	0.081*
10–99 employees (ref ≥ 100)	10.6	− 21.7 to − 42.9	0.52	1.10	− 36.1 to 38.3	0.95
Contractual hours	− 1.1	− 2.8 to 0.6	0.19*	0.98	− 0.77 to 2.7	0.27
Working hours	− 1.36	− 2.6 to − 0.1	0.03*	− 0.4	− 1.8 to 1.0	0.55
Job type						
Executive (ref = Policy)	34.9	− 11.8 to 81.6	0.14*	17.0	− 38.1 to 72.0	0.54
Administrative (ref = Policy)	− 6.5	− 62.9 to 50.0	0.82	− 18.5	− 86.6 to 49.6	0.59
Advisory (ref = Policy)	− 11.1	− 92.9 to 70.7	0.79	− 17.1	− 92.5 to 58.4	0.65
Management (ref = Policy)	32.1	− 25.3 to 89.5	0.27	18.7	− 48.7 to 86.2	0.58
Tasks						
Physical (ref = mental)	35.7	− 2.7 to − 74.1	0.068*	57.5	15.5 to 99.4	0.008*
Both (ref = mental)	31.7	− 2.5 to 65.8	0.069*	54.0	19.2 to 88.8	0.003*
Physical working conditions						
Work demands						
Standing (no = ref)	27.1	− 0.22 to 54.5	0.05*	14.9	− 17.1 to 47.0	0.36
Sitting (no = ref)	− 23.7	− 50.7 to 3.2	0.084*	− 27.5	− 60.2 to 5.3	0.10*
Walking (no = ref)	41.3	12.7 to 69.9	0.005*	14.5	− 18.8 to 47.8	0.39
Kneeling/squatting (no = ref)	9.6	− 22.3 to 41.4	0.55	67.9	33.4 to 102.5	0.00*
Difficulty standing (no = ref)	7.3	− 27.3 to 41.9	0.68	10.8	− 26.8 to 48.4	0.57
Difficulty sitting (no = ref)	− 24.1	− 52.1 to 4.0	0.092*	− 19.4	− 52.1 to 13.3	0.24
Difficulty moving > 5 kg (no = ref)	− 0.12	− 28.7 − 28.5	0.99	13.7	− 22.6 to 49.9	0.45
Difficulty moving > 20 kg (no = ref)	− 34.9	− 75.4 to 5.6	0.091*	− 7.4	− 58.2 to 43.5	0.77
Difficulty using power with arm/hand (no = ref)	14.1	− 26.1 to 54.3	0.49	− 8.5	− 53.1 to 36.1	0.71
Difficulty using vibrating/tamping work tools (no = ref)	0.77	− 32.1 to 33.6	0.96	8.0	− 34.0 to 50.0	0.71
Difficulty driving (no = ref)	− 26.8	− 64.1 to 10.3	0.16*	8.4	− 28.2 to 45.0	0.65
Difficulty working in uncomfortable position (no = ref)	− 18.1	− 49.8 to 13.7	0.26	− 37.4	− 74.8 to 0.05	0.05*
Difficulty w lengthy work in same position (no = ref)	− 13.6	− 42.4 to 15.3	0.35	− 37.8	− 69.4 to 6.2	0.02*
Psychosocial working conditions						
Quantitative demands	− 3.8	− 13.0 to 5.4	0.41	6.7	− 1.6 to 14.9	0.11*
Tempo work pace	− 1.0	− 9.1 to 7.0	0.80	6.2	− 2.9 to 15.2	0.18*
Emotional demands	− 0.28	− 7.1 to 6.5	0.94	3.5	− 5.8 to 12.8	0.50
Influence at work	− 10.3	− 18.1 to − 2.4	0.011*	− 2.5	− 9.5 to 4.5	0.48
Possibilities for development	− 13.8	− 23.3 to − 4.4	0.004*	− 10.0	− 20.0 to − 0.0	0.05*
Meaning of work	− 7.2	− 18.7 to 4.5	0.23	3.3	− 6.3 to 12.9	0.49
Commitment to workplace	− 3.4	− 12.1 to 5.4	0.45	− 2.63	− 12.8 to 7.6	0.61
Predictability	− 10.0	− 18.3 to − 1.8	0.02*	− 6.0	− 15.3 to 3.4	0.21
Recognition	− 7.5	− 17.0 to 1.9	0.12*	− 0.9	− 10.0 to 8.3	0.85
Role clarity	2.9	− 8.8 to − 14.7	0.62	− 0.8	− 13.0 to 11.5	0.90
Quality of leadership	1.1	− 7.9 to − 10.1	0.81	− 8.5	− 17.9 to 1.0	0.08*
Social support of supervisor	− 3.4	− 12.4 to 5.7	0.46	− 4.4	− 12.9 to 4.1	0.31
Social support of colleagues	− 2.3	− 12.3 to 7.8	0.65	− 2.3	− 11.5 to 7.0	0.63
Work adjustments						
Work adjustments (% out of 12)	1.5	− 0.84 to 3.9	0.21	− 0.003	− 0.008 to − 0.002	0.29
Work adjustments (no = ref)	64.6	− 44.2 to 173.5	0.23	− 26.8	− 227.2 to 173.6	0.77

Adjusted for age, sex, education, and number of comorbidities. *P ≤ 0.20
